# Safety and Feasibility of Intradiscal Administration of Matrilin-3-Primed Adipose-Derived Mesenchymal Stromal Cell Spheroids for Chronic Discogenic Low Back Pain: Phase 1 Clinical Trial

**DOI:** 10.3390/ijms242316827

**Published:** 2023-11-27

**Authors:** Dong Hyun Lee, Kwang-Sook Park, Hae Eun Shin, Sung Bum Kim, Hyejeong Choi, Seong Bae An, Hyemin Choi, Joo Pyung Kim, Inbo Han

**Affiliations:** 1 Department of Neurosurgery, Spine Center, The Leon Wiltse Memorial Hospital, Suwon 16480, Republic of Korea; whatdj@naver.com; 2 Department of Neurosurgery, CHA Bundang Medical Center, CHA University, Seongnam-si 13496, Republic of Korea; 3 Department of Neurosurgery, Kyung Hee University, Seoul 02447, Republic of Korea; 4 Department of Radiology, CHA Bundang Medical Center, CHA University, Seongnam-si 13496, Republic of Korea

**Keywords:** intervertebral disc, degeneration, matrilin-3, adipose-derived stromal cell, spheroid, hyaluronic acid

## Abstract

Functionally enhanced mesenchymal stromal cells participate in the repair of intervertebral disc. This study aimed to assess the safety and tolerability of intradiscal administration of matrilin-3-primed adipose-derived stromal cell (ASC) spheroids with hyaluronic acid (HA) in patients with chronic discogenic low back pain (LBP). In this single-arm, open-label phase I clinical trial, eight patients with chronic discogenic LBP were observed over 6 months. Each patient underwent a one-time intradiscal injection of 1 mL of 6.0 × 10^6^ cells/disc combined with HA under real-time fluoroscopic guidance. Safety and feasibility were gauged using Visual Analogue Scale (VAS) pain and Oswestry Disability Index (ODI) scores and magnetic resonance imaging. All participants remained in the trial, with no reported adverse events linked to the procedure or stem cells. A successful outcome-marked by a minimum 2-point improvement in the VAS pain score and a 10-point improvement in ODI score from the start were observed in six participants. Although the modified Pfirrmann grade remained consistent across all participants, radiological improvements were evident in four patients. Specifically, two patients exhibited reduced high-intensity zones while another two demonstrated decreased disc protrusion. In conclusion, the intradiscal application of matrilin-3-primed ASC spheroids with HA is a safe and feasible treatment option for chronic discogenic LBP.

## 1. Introduction

Chronic discogenic low back pain (LBP), resulting from intervertebral disc (IVD) degeneration, is a leading cause of disability with significant socio-economic consequences and remains a challenging condition to address [1,2,3]. Currently, there is no established clinical therapy that effectively addresses and reverses IVD degeneration. Treatment modalities for discogenic primarily consists of conservative treatments and image-guided minimally invasive percutaneous interventions. Despite advances in conservative and minimally invasive percutaneous treatments, a notable portion of patients continue to experience persistent LBP, emphasizing the need for innovative therapeutic approaches [4,5,6,7]. In recent years, the emergence of cell therapy strategies and the rapid expansion of regenerative medicine research have led to significant advances in this field [3,8,9,10,11,12,13,14]. Among these, mesenchymal stromal cells (MSCs) have been recognized in regenerative medicine as a promising cellular treatment for discogenic LBP [15,16,17,18,19,20,21]. To date, including our own research, ten clinical studies investigating intradiscal MSCs injections for discogenic LBP have been published in the scientific literatures [22]. In our prior clinical trial, we demonstrated the safety and tolerability of co-administering autologous adipose-derived stromal cells (ASCs) and hyaluronic acid (HA) in patients suffering from chronic discogenic LBP [21]. Remarkably, of the 10 participants, six experienced significant improvements in both Visual Analogue Scale (VAS) pain and Oswestry Disability Index (ODI) scores. Within this 12-month study span, three of the improved participants also showcased disc rehydration, as measured by the apparent diffusion coefficient, alongside sustained pain alleviation.

Although clinical success is promising, the effectiveness of stem cell regenerative therapy is still not well-defined. Furthermore, the attainment of optimal therapeutic effects is complex due to the detrimental conditions associated with IVD degeneration, which include inflammation, acidity, and low oxygen levels. Consequently, contemporary research aims to enhance the inherent capabilities of MSCs. Various methods have been developed to increase the therapeutic potential of these cells [17,18,19]. One such approach involves cultivating MSCs in hypoxic conditions, which has been shown to bolster their regenerative functions [23,24]. Additionally, the use of biomaterials serves not only as a delivery mechanism for MSCs but also demonstrates synergistic benefits in restoration of degenerated disc [25,26]. Research both in vitro and in vivo has underscored the benefits of priming MSCs with proteins such as TGF-β, suggesting a notable improvement in their ability to repair tissues [10]. Moreover, spheroids, which are clusters of tightly packed cells, overcome many of the difficulties found with transplanting individual cells. Aggregating cells into spheroids not only improves their survival but also enhances their ability to support new blood vessel formation, diminish inflammation, and promote the repair and regeneration of tissue, compared to individual cells [27]. Several methods for the formation of ASC spheroids have been developed, including the use of ultra-low adhesion flasks, hanging drop techniques, spinner flasks, and innovative tools such as lockyballs (microscaffolds featuring a porous wall with interlocking hooks), GMP techniques utilizing microwell plates with gelatin microparticles, and various scaffold-free approaches [28].

**Figure 1:**
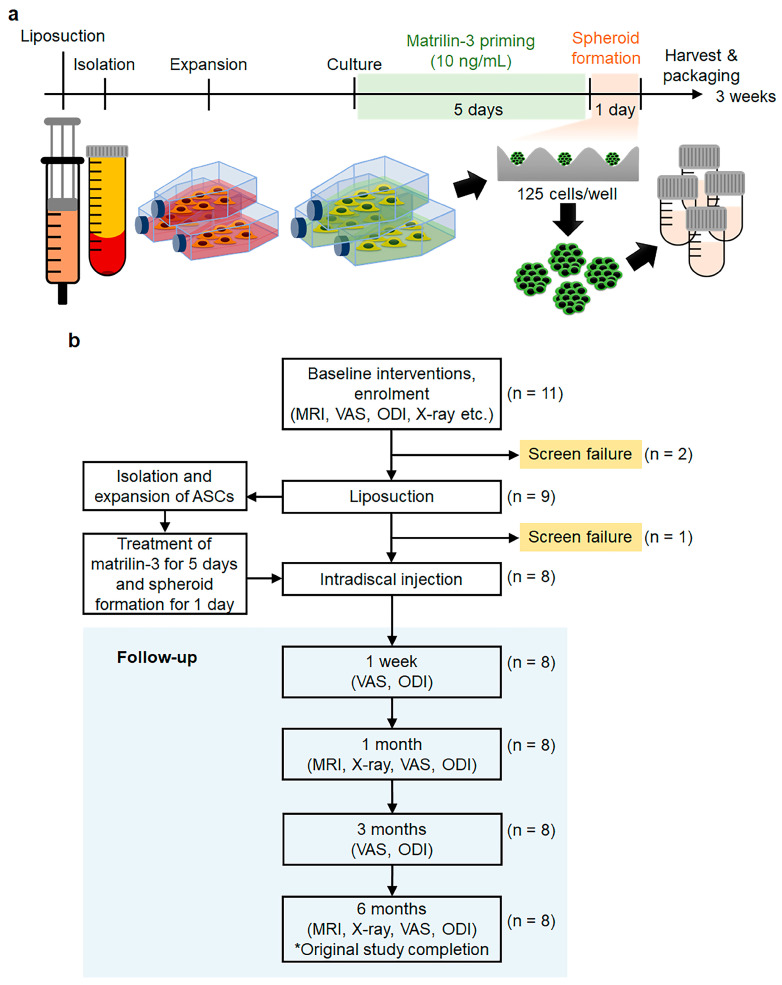
(**a**) Schematic design of production process for autologous adipose-derived stem cell (ASC) spheroids primed with matrilin-3 and (**b**) flow chart depicting the key steps and stages of this clinical study.

In our previous studies aimed at enhancing the functionality of ASCs through protein priming, we identified matrilin-3 as a significant enhancer of ASCs’ therapeutic efficacy in cartilage regeneration. This was demonstrated in both in vitro and in vivo experiments, where matrilin-3 also showed its potential to counteract hypertrophy [29,30]. Specifically, matrilin-3-primed ASC spheroids exhibited enhanced cartilage markers and suppressed chondrocyte hypertrophy during differentiation. Furthermore, these matrilin-3-primed ASC spheroids restored the expression of nucleus pulposus (NP)-related extracellular matrix components and decreased hypertrophic markers in degenerated NP cells [29]. We determined the optimal conditions, including the optimal concentration of matrilin-3 and the ideal culture duration, for the formation of spheroids from ASCs as shown in Figure 1. Based on these observations, we hypothesized that autologous matrilin-3 primed ASC spheroids could potentially address degenerated disc conditions and relieve the resultant chronic LBP.

MSC transplantation in degenerative IVDs may lead to unwanted osteophyte formation as a consequence of cell leakage. To minimize this risk, it has been proposed to combine MSCs with cell carriers such as HA during transplantation, which could potentially decrease the likelihood of osteophyte formation [19,31]. To test this in this clinical trial, we obtained adipose tissue from the abdominal region of patients suffering from chronic discogenic LBP. From this tissue, we isolated ASCs and exposed them to 10 ng/mL of matrilin-3 for five days. Subsequently, we generated spheroids with a density of 125 cells/well over an additional day (Figure 1). This phase 1 clinical trial then introduced these matrilin-3 primed ASC spheroids, combined with HA, to the patients with chronic discogenic LBP, those who had not responded to conventional conservative treatments. Our primary objective was to determine the safety and feasibility of this approach using tools VAS, ODI, and MRI evaluations.

## 2. Results

### 2.1. Patient Characteristics

Of the 11 patients who initially participated, eight successfully completed the study. During the screening phase, two were excluded because they did not produce a sufficient number of ASCs in culture. At the 6-month follow-up, all remaining participants had finished the study (Figure 1). Table 1 provides a detailed overview of the participants’ demographics and baseline attributes at the onset of the study.

The study involved eight participants: seven male and one female, with ages ranging between 32 and 64 years (mean: 48.4 years). The duration of chronic LBP symptoms ranged from 11 to 96 months (mean: 46.6 months). Out of these participants, five participants (cases 2, 3, 4, 5, and 7) were categorized as overweight based on their body mass index (BMI: 25–29.9 kg/m^2^). None were classified as obese (BMI ≥ 30 kg/m^2^), and the remaining three (cases 1, 6, and 8) had a BMI within the normal range (18.5–24.9 kg/m^2^).

Prior to the intradiscal injection, each vial contained 1.0 × 10^7^ matrilin-3 primed ASCs. The number of Spheroids with matrilin-3 priming was 1.0 × 10^5^ cells/vial. Every participant received an implantation of 600 μL matrilin-3-primed ASC spheroids (6.0 × 10^6^ cells) amalgamated with 400 μL of HA.

In cases 2, 3, 4, 5, and 7, the ASCs chiefly displayed MSC-specific markers, CD44 and CD73, with an average prevalence of 99.6% and 97.0%, respectively. In contrast, case 1 showed a reduced CD44 prevalence (87.2%) but maintained a high CD73 rate (97.9%). For case 6, CD44 expression was at a full 100%, yet CD73 was slightly lower at 87.4%. Notably, all ASCs from the participants presented an almost negligible expression of CD45, with an average occurrence of roughly 0.9% (Table 1).

### 2.2. Clinical Outcomes

#### 2.2.1. Safety Variables

Regarding safety measures, all patients were discharged 4 h post-implantation. Throughout the treatment phase and up to the 6-month follow-up, there were no reported adverse events (AEs) or serious adverse events (SAEs) linked to the procedure. Peripheral blood tests were conducted at intervals of 1 week and at 1, 3, and 6 months after the intervention. The results from these tests showed no notable anomalies, further affirming the procedure’s safety.

#### 2.2.2. Outcome of VAS for Pain and ODI Scores

**Figure 2:**
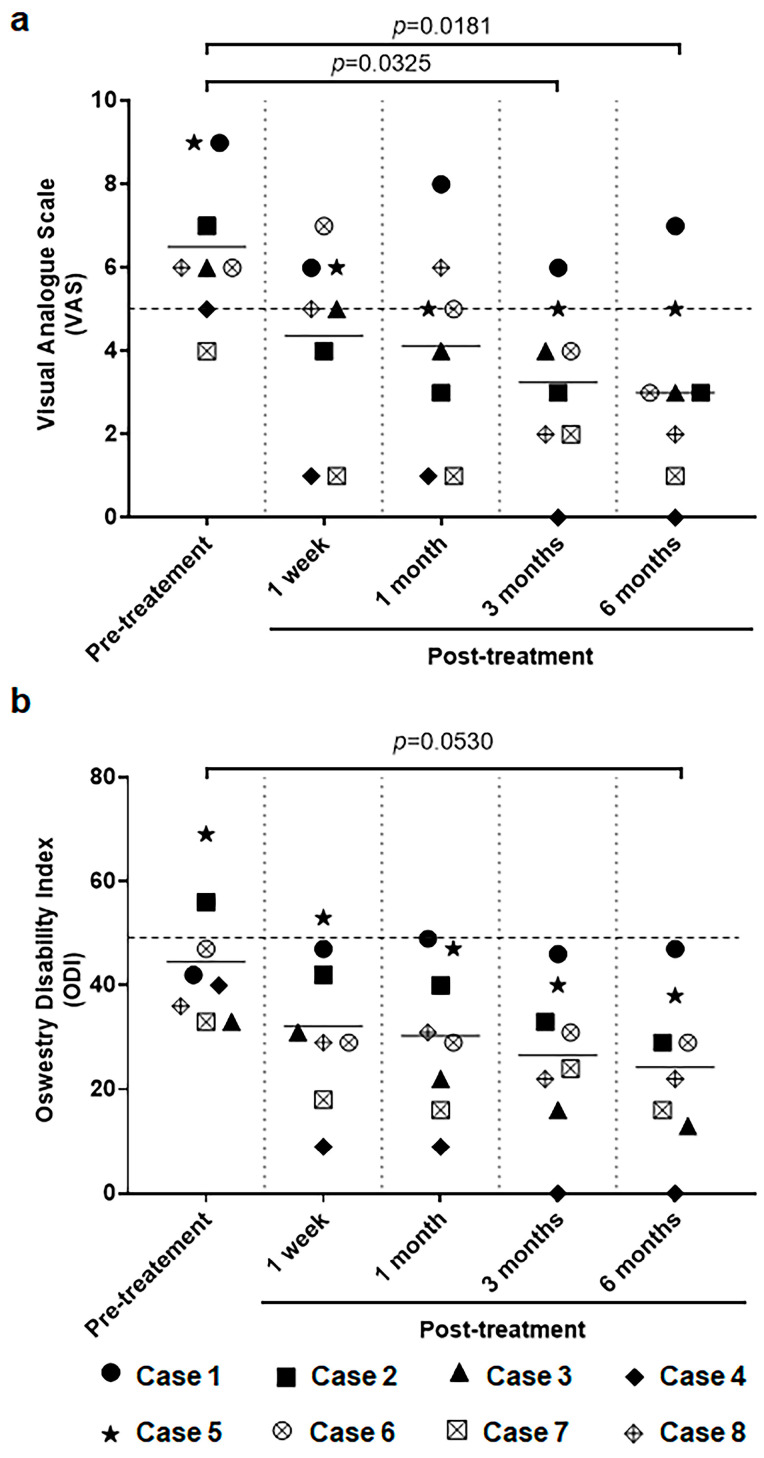
Changes in pain levels measured using the Visual Analog Scale (VAS) (**a**) and functional disability assessed by the Oswestry Disability Index (ODI) (**b**). Among the patients, six (Cases 2, 3, 4, 5, 7, and 8) demonstrated a substantial and clinically significant reduction in both VAS and ODI scores, with an improvement of at least 10 points for ODI and 2 points for VAS compared to pretreatment levels at the 6-month follow-up.

**Figure 3:**
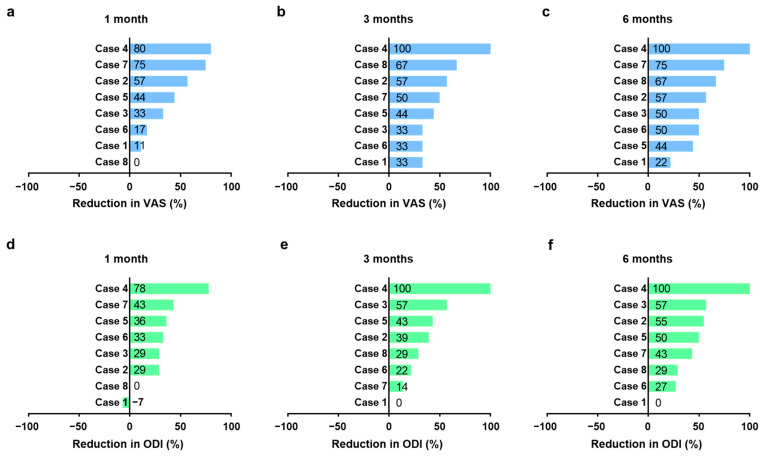
Tornado plots to visualize the reduction rates in Visual Analog Scale (VAS) (**a**–**c**) and Oswestry Disability Index (ODI) scores (**d**–**f**) at 1, 3, and 6 months, as well as the changes in T2 values at 1 and 6 months.

Secondary endpoints encompassed changes in baseline VAS and ODI scores. The criteria for treatment success were delineated as a decrease of at least 2 points in the VAS score for pain and a reduction of 10 points or greater in the ODI score compared to pre-treatment baseline values [32]. At the 6-month follow-up, both VAS and ODI scores demonstrated marked improvement in six of the eight participants (specifically, cases 2, 3, 4, 5, 7, and 8) (Table 2 and Table 3, Figure 2 and Figure 3). Consequently, the intervention was deemed efficacious for these six participants. In case 6, although the outcomes did not satisfy the pre-established efficacy thresholds, noteworthy alterations were evident. The ODI score for case 6 reflected a modest improvement from 45% to 33%, while the VAS pain score exhibited a considerable reduction, descending from 6 to 3. In contrast, case 1 demonstrated a slight decrease in the VAS score, moving from 9 to 7, yet the ODI score remained stagnant. (Table 2 and Table 3, Figure 2 and Figure 3).

#### 2.2.3. Radiological Outcomes

**Figure 4:**
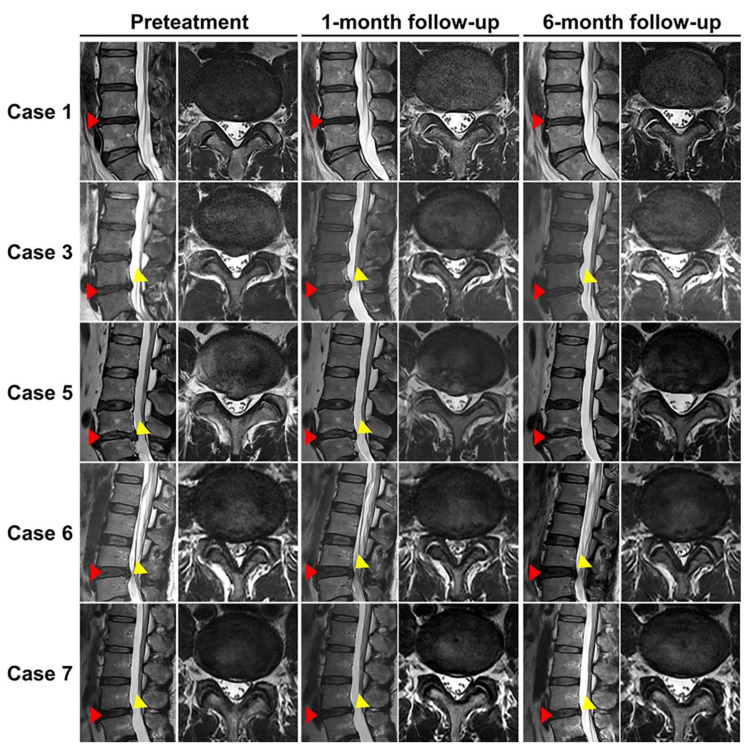
Assessment of disc degeneration grade based on the evaluation of sagittal and axial T2-weighted images. Red triangle indicates the treated L4-5 disc. Yellow triangle indicates disc herniation.

Six months post-implantation, subsequent X-rays and MRI scans showed no reduction in the height of the treated discs. Moreover, there were no Modic changes detected in the lumbar endplates among any of the participants. At the beginning of the study, every participant displayed degenerative IVDs, rated at a minimum of a modified Pfirrmann grade III. By the six-month mark, the conditions of these discs appeared stable, as indicated by unchanged modified Pfirrmann grades for all participants (Table 4 and Figure 4). Radiological improvements were evident in Cases 1, 3, 5, and 7. For instance, HIZ, a known contributor to chronic LBP, was no longer present in Cases 1 and 7 according to the six-month MRI [33]. MRI evaluations at the same interval revealed a marked decrease in disc protrusion. Importantly, we observed no evidence of osteophyte development or IVD reherniation.

## 3. Discussion

The range of image-guided percutaneous interventional techniques developed for the treatment of discogenic LBP emphasizes minimal invasiveness and maximal pain relief. These procedures, which include radiofrequency annuloplasty, intradiscal electrothermal therapy, and chemonucleolysis with agents like medical ozone, operate through distinct mechanisms. However, the evidence from various studies remains inconclusive regarding their effectiveness. The ongoing debate highlights the need for more definitive research to establish the safety and efficacy of stem cell therapy and its potential to be preferred alternatives to percutaneous interventional techniques [34]. Stem cell therapy for chronic discogenic LBP has been extensively explored in clinical trials [16,18,19,21,35,36,37,38,39]. Table 5 summarizes the results of clinical trials using stem cells to address discogenic LBP [16,35,37,38,39,40,41,42,43,44,45,46,47]. A study with 10 patients treated with autologous ASCs (10 × 10^6^ cells/disc) showed a 68% 62.4% decrease in VAS and ODI scores, respectively, at 6 months, with consistent benefits at 12 months with increase in water content on MRI [16]. Mochida et al., found that autologous bone marrow-derived MSCs, co-cultured with NP cells and transplanted into degenerated discs, improved Japanese Orthopaedic Association (JOA) scores significantly over 36 months without notable radiographic changes [41]. Elabd et al. reported long-term improvements in quality of life and physical function in five patients treated with MSCs (15 to 51.6 × 10^6^ cells) derived from bone marrow after hypoxic culture [42]. Another study involving 33 patients who received various doses (average 2.3 × 10^6^, range 1.72–4.5 × 10^6^) of MSCs reported a 55% pain reduction maintained from 3 months post-treatment and Functional Rating Index (FRI) improvements. In addition, disc bulge reduction significantly correlated with pain improvement in 8 of these 20 patients [43]. Pettine et al. observed substantial improvements in VAS and ODI scores in 26 patients who were candidates for spinal fusion or disc replacement as soon as 3 months post-treatment with autologous MSCs (121 × 10^6^), which persisted for 36 months. However, 6 out of the 26 patients required surgery during the study. Noteworthy is the finding that higher MSC doses correlated with significant ODI and VAS score improvements and at least a one-grade enhancement in the Pfirrmann score for some patients over a year [40,44,45].

What sets our clinical trial apart is the use of matrilin-3 protein to enhance MSC functionality before processing them into spheroids for administration. Matrilin-3 plays a crucial role in the development of cartilage and ossification, and as well as interacting with essential growth factors [29,30,48,49]. It enhances the expression of collagen II and aggrecan, key components for maintaining cartilage and IVD integrity. Additionally, matrilin-3 mitigates inflammation and matrix degradation. This is particularly significant given that cells cultured in spheroids show improved survival and enhanced chondrogenic potential [27,29,48,50].

Our in vitro studies revealed that a 5-day priming with 10 ng/mL of matrilin-3 led to maximum mRNA expression of type 2 collagen and aggrecan [48]. Moreover, ASC spheroids formed at specific densities released beneficial growth factors while reducing the release of certain hypertrophic ECM components. In a rabbit model of lumbar disc degeneration, these matrilin-3-primed ASC spheroids led to improved tissue restoration [48]. Determining the appropriate sample size for a Phase I clinical trial can be challenging. However, drawing from existing literature, we believed that a cohort of 8 patients would be adequate to fulfill the objectives of our trial, especially since typical Phase I clinical trials have sample sizes ranging from six to ten participants [51]. Thus, our Phase I clinical trial, which included 8 patients based on common sample sizes for this phase [51], primarily aimed to assess the safety and efficacy of using matrilin-3 primed ASC spheroids combined with HA in treating chronic discogenic LBP. Six months post-transplantation, six (specifically cases 2, 3, 4, 5, 7, and 8) out of the eight patients showed significant improvement in pain and functionality. Significantly, out of these six patients, MRI results (Figure 4) showed improvements in either HIZ or disc protrusion for three of them, specifically cases 3, 5, and 7. Current studies indicate that most lumbar disc herniations, except for notably large ones, generally remain stable and show minimal changes over a span of four years [52]. Cases 3 and 5 in our study, which initially presented with focal disc protrusions, exhibited a marked reduction post-treatment, suggesting potential efficacy of the treatment for such conditions (Figure 4) [53]. Initially, T2-weighted MRI images (Figure 4) revealed that both cases 1 and 7 had a HIZ located in the posterior annulus of the L4/5 discs. From a histological perspective, an HIZ lesion is frequently linked to the development of granulation tissue in the outer portion of the annulus fibrosus. This lesion is generally considered a reliable indicator of painful disruptions in the outer annulus [54,55,56]. For case 7, both the VAS and ODI scores showed notable enhancements, alongside a pronounced decrease in the HIZ of the treated disc. While case 1 only displayed a modest decrease of 2 points in the VAS (from 9 to 7) with no noticeable change in the ODI score, categorizing it as a treatment non-responder, there was a pronounced decrease in the HIZ. The notable HIZ reduction, coupled with the clinical improvement seen in case 7 after administering matrilin-3-primed ASC spheroids intradiscally, points to potential therapeutic benefits.

Although the exact mechanism of improvement remains elusive, our previous studies hint that matrilin-3-primed ASC spheroids could effectively restore ECM in degenerated disc, with benefits attributed to spheroid formation and matrilin-3 priming [48,57]. Matrilin-3 priming may promote the capacity to mitigate inflammatory cytokines and stimulate ECM production through the secretion of multiple regenerative factors [30,48]. Spheroid-based cell implantation significantly enhances NP regeneration compared to implanting individual cells, largely due to interactions between N-cadherin and integrin-β1 during spheroid formation, thus promoting ECM synthesis [57]. Hence, matrilin-3 primed ASC spheroids could offer a viable therapeutic approach for disc regeneration by leveraging the combined advantages of differentiation and spheroid formation.

In our study, we examined reasons for treatment failures in cases 1 and 6. Case 1 showed a significant drop in CD44 positivity (87.2%), potentially affecting the efficacy of the matrilin-3-primed ASC spheroids, while maintaining high CD73 positivity (97.9%). In contrast, case 6, despite having full CD44 positivity (100%), displayed decreased CD73 positivity (87.4%) and presented radiological signs of degenerative spondylolisthesis and spinal stenosis. These factors might have influenced the treatment’s ineffectiveness (Figure 4).

Our study aligns with previous clinical trials [16,35,37,38,39,40,46,47] in utilizing intradiscal injections. We assessed validity using VAS and ODI. Unlike prior studies, our selection of discs for cell administration was based on MRI criteria rather than discography, to avoid potential disc damage from needle puncture [58]. Our approach involved the use of matrilin-3-primed ASCs in treating discogenic LBP, a method not yet explored in existing clinical trials. Additionally, we employed cell spheroids to enhance efficacy. Notably, this constitutes the inaugural phase 1 clinical trial to use matrilin-3-primed ASC spheroids in patients suffering from chronic discogenic LBP.

As we extend our research, our phase 2 clinical trial will aim to build upon these initial findings. In this next phase, we intend to introduce a control group and enlarge our participant pool, enhancing the statistical robustness of our results. Additionally, we plan to extend the follow-up duration to more effectively assess the long-term efficacy of matrilin-3-primed ASC spheroids.

Our study acknowledges multiple limitations: a limited sample size, the lack of a control group, and a brief 6-month follow-up period. While discography might precisely pinpoint the disc causing chronic LBP, our selection for cell injection was based on clinical observations, MRI findings, and pain block procedures. This choice brings into question the exactness in identifying the chronic LBP source. The HA, used alongside the ASC spheroid implantation to prevent cell leakage, was originally designed for knee osteoarthritis, not for disc interventions. Given these limitations and the acknowledgment that additional comprehensive research is needed, it’s prudent to be cautious when drawing definitive conclusions regarding the long-term safety and efficacy of the treatment. Nevertheless, our initial findings point to the safety and potential therapeutic value of our approach.

## 4. Materials and Methods

### 4.1. Study Design

The study protocol received approval from the Institutional Review Boards and Ethical Committees at CHA Bundang Medical Center (approval number 2018-10-013-034, October 2018) and the Ministry of Food and Drug Safety of Republic of Korea (approval number 202100348, March 2021). The study rigorously followed the principles stated in the Declaration of Helsinki and adhered to the guidelines of good clinical practice (ISO 14155). This clinical trial was registered with the ClinicalTrials.gov database under the identifier: NCT05011474. All study participants gave their written informed consent. The study was designed as a single-arm, open-label, phase I clinical trial carried out at CHA Bundang Medical Center with a follow-up duration of 6 months. We used a range of metrics to assess treatment outcomes, including the VAS, ODI, and lumbar spine MRI. The assessment schedule for the study is provided in Table 6. During our recruitment phase from April 2021 to July 2022, we screened 11 patients suffering from chronic LBP, with diagnoses based on clinical and neuroimaging standards.

Our primary goals centered on evaluating the safety and tolerability of intradiscal administration of matrilin-3 primed ASC spheroids combined with HA. We determined this through physical and neurological examinations, recording of adverse events (AEs), and observing shifts in laboratory values. Regarding treatment results, we noted changes in patient-reported VAS and ODI scores, as well as MRI findings. Additionally, our study protocol included follow-up appointments post-treatment at intervals of 1 week, and then at 1, 3, and 6 months, ensuring thorough clinical, laboratory, and imaging assessments. Regarding sample size estimation, Phase 1 trials do not require formal calculations as needed in Phase 2 and 3 studies. Given that standard Phase 1 clinical trials commonly include 6–10 participants, we determined that enrolling 8 subjects will sufficiently meet the objectives of our trial [51].

### 4.2. Patient Selection

Chronic discogenic LBP was diagnosed based on a combination of clinical symptoms and MRI findings [59,60]. Criteria for inclusion and exclusion are presented in Table 7 were set to guide patient selection. Eligible participants included men and women between the ages of 18 and 70, who had experienced symptoms of axial chronic discogenic LBP for a minimum of 3 months, with an intensity rating of at least 4/10 on the VAS. Additionally, they needed to have a disability score of at least 30% on the ODI and had shown inadequate relief after 3 months of standard conservative treatments, including medication, intensive physical therapy, and local anesthetic injections in the facet joints or medial branches. The presence of moderate IVD degeneration (modified Pfirrmann grade III–VII as identified on T2-weighted MRI) and a retention of more than 50% disc height were also mandatory for inclusion [60,61]. The exclusion criteria included pregnancy or breastfeeding; prior lumbosacral surgeries, severely herniated disc, or spinal stenosis requiring surgical intervention; Modic type 3 change; MRI-evident spinal infection; and uncontrolled hypertension, uncontrolled diabetes, and other systemic diseases including cancer, autoimmune diseases, hematological diseases, and kidney disease (Table 2). All participants underwent a medial branch block to eliminate the possibility of facet joint pain being the primary source of their chronic LBP.

### 4.3. Diagnosis of Discogenic LBP and Selection of Disc for Injection

Candidates who met the general eligibility criteria and reported a history of chronic LBP underwent initial evaluations for study inclusion. Initially, we identified potential lumbar levels of concern based on clinical symptoms and MRI evidence of IVD degeneration. Such evidence encompassed features like the high-intensity zone (HIZ), disc protrusion, or endplate Modic changes [62,63,64,65]. However, it was vital to rule out chronic LBP stemming from the lumbar facet joints or surrounding soft tissues. D.H.L. (Spine surgeon, expert for minimal invasive spine surgery) performed a local anesthetic block on the medial branch of the dorsal ramus that supplies the facet joint to determine whether the chronic LBP originated from the facet joint. While discography is a potential diagnostic tool for discogenic LBP, we chose not to utilize it due to concerns about its reliability in accurately identifying the origin of pain. Additionally, the needle puncture associated with the procedure could potentially worsen disc damage [58]. Instead, we selected discs for cell administration based on MRI indicators such as degree of disc degeneration, HIZ, disc protrusion, and diminished disc height.

### 4.4. Primary and Secondary end Points

The primary endpoints were the safety and tolerability of administering matrilin-3-primed ASC spheroids combined with HA to patients with chronic LBP. Comprehensive safety evaluations were performed, encompassing peripheral blood tests, vital sign measurements, physical and neurological assessments, and systemic monitoring. Any AEs and SAEs were diligently recorded throughout the treatment and subsequent follow-up phases. The secondary endpoints included changes in the VAS, ODI, as well as variations in disc degeneration from the baseline to 6 months post-treatment. Scheduled clinical assessments and laboratory tests were conducted at specific intervals: 1 week, 1 month, 3 months, and 6 months after treatment. To evaluate changes in the disc height and hydration, lumbar spine X-ray imaging and T2-weighted MRI scans were performed before the procedure and at the 1- and 6-month marks post-treatment.

### 4.5. MRI Acquisition

All participants underwent lumbar spine MRI scans using a 3.0-T magnetic resonance scanner (Signa HDxt; GE Medical Systems, Milwaukee, WI, USA), adhering to a previously defined protocol [16]. We utilized sagittal plane T2-weighted imaging and axial plane imaging to gauge the degree of IVD degeneration. The parameters for T2-weighted imaging were set as follows: repetition time of 3200 ms, echo time of 121.3 ms, number of excitations set to 2, slice thickness of 3 mm, and an interslice gap of 0.33 mm. An experienced radiologist (co-author: H.C.), who was blinded to the patient information, performed a meticulous analysis of all the scans. The level of IVD degeneration was assessed using the modified Pfirrmann grading system, which categorizes degeneration on a scale from grade I (indicating a normal disc) to grade VIII (indicating severe degeneration with disc collapse) [43,60,61,66].

### 4.6. Cell Production and Preparation

Three weeks prior to transplantation, subcutaneous abdominal adipose tissues were obtained using liposuction. This procedure was executed by a skilled plastic surgeon in the operating room, using local anesthesia. The liposuction yielded an average volume of 176 mL, with a range from 140 mL to 250 mL. Following the procedure, patients were observed for 4 h before discharge. The harvested liposuction samples were transported under controlled conditions to the GMP facility of CHA Biotech Co., Ltd. (Pangyo, Seongnam, Republic of Korea). The lipoaspirate underwent aseptic processing using established methodologies [30,48]. The adipose samples were twice rinsed with Dulbecco’s phosphate-buffered saline containing 2% gentamycin (Gibco BRL, Grand Island, NY, USA). They were subsequently treated with an equal volume of 0.1% collagenase (Sigma-Aldrich, St. Louis, MO, USA) for an hour. Post-digestion, centrifugation was performed, and the samples were triple-filtered using 100-μm strainers before seeding onto T75 flasks. Isolated ASCs were cultured in low-glucose Dulbecco modified Eagle medium (DMEM; HyClone, GE Healthcare Life Sciences, Logan, UT, USA), fortified with 10% fetal bovine serum (FBS; HyClone), 50 μg/mL gentamicin, and 10 ng/mL human basic fibroblast growth factor (Gibco BRL). They were maintained at 37 °C within a 5% CO_2_ environment. To validate the ASCs, we employed the Trypan blue technique for viability and flow cytometry (CytoFLEX; Beckman Coulter, Indianapolis, IN, USA) to assess cell surface markers, ensuring positivity for CD44 and CD73 and negativity for CD45. ASCs were then plated at a density of 1500 cells/cm^2^.

After 12 h, the adhered cells were subjected to a 12-h serum starvation phase, followed by cultivation in DMEM supplemented with 10% FBS and 10 ng/mL recombinant matrilin-3 (Aviva Systems Biology, San Diego, CA, USA) for 5 days. The medium, including matrilin-3, was changed daily. The pretreated cells were subsequently cultured at a density of 3.0 × 10^4^ cells/cm^2^ for 24 h on EZSPHERE plates (125 cells/well; AGC Techno Glass Inc., Shizuoka, Japan) to enable spheroid formation (Figure 1).

The formed ASC spheroids were gathered, vial-packaged, and meticulously examined for the absence of pathogens like viruses, bacteria, mycoplasma, and endotoxins. For transplantation, autologous ASC spheroids primed with matrilin-3 were transported to the operating room, standardized at 1.0 × 10^7^ cells/mL/vial.

### 4.7. Preparation of HA (Hyruan Plus^®^) for Cell Delivery

Hyruan Plus^®^ (LG Life Sciences, Iksan, Republic of Korea) is a transparent and viscous gel comprised of cross-linked HA, containing sodium hyaluronate at a concentration of 20 mg (1%) in a 2.0 mL syringe. Its HA component is derived through microbial fermentation of the bacterium Streptococcus zooepidermicus. Distributed in over 25 countries, Hyruan Plus^®^ is primarily recommended for treating osteoarthritis in various joints, including the knee, shoulder, hip, and ankle. For the purposes of this clinical trial, the use of Hyruan Plus^®^ for cell delivery was approved by Republic of Korea’s MFDS.

### 4.8. Implantation of Matrilin-3 Primed ASC Spheroids in Combination with HA

Intervertebral disc displaying signs of chronic LBP and confirmed IVD degeneration through T2-weighted MRI were chosen for implantation with a combination of matrilin-3 primed ASC spheroids and HA. Our selection relied on radiographic data and pain-blocking methods, such as the medial branch block. For the cell injection, patients were placed in a prone position. The injection site was anesthetized using 1% buffered lidocaine. The matrilin-3 primed ASC spheroids (1.0 × 10^7^ cells/mL/vial) and HA (Hyruan Plus^®^) were transported from our GMP facility (CHA Biotec Co., Ltd., Pankyo, Republic of Korea) directly to the operating room. A 22-gauge spinal needle was used to inject the mix of matrilin-3 primed ASC spheroids (600 μL, 6 × 10^6^ cells) and HA (400 μL, of Hyruan Plus^®^) precisely into the center of the targeted disc. Each patient consistently received an injection volume of 1 mL, guided by C-arm fluoroscope guidance. To ensure retention of the injected material, the spinal needle was held in place for additional 5 min post-injection. After the procedure, patients received a three-day pain medication regimen and were recommended to limit physical activity for two weeks. The implantation of matrilin-3 primed ASC spheroids was carried out by I.H., a spine surgeon specializing with expertise in translational research.

### 4.9. Statistical Analysis

The individual responsible for data analysis was kept unaware of the group assignments. The primary endpoint assessed was the treatment effect at each follow-up interval. Both pre- and post-treatment data were compared statistically using the Wilcoxon signed-rank test and the paired *t*-test for the VAS and ODI. The data are presented as mean ± standard deviation.

Differences between patients were assessed via analysis of variance. Subsequent post-hoc tests were performed to delve deeper into these variations. A *p*-value below 0.05 was deemed statistically significant. All statistical analyses were conducted using SPSS version 26 (IBM Corp., Armonk, NY, USA).

## 5. Conclusions

Our clinical study confirmed the safety and tolerability of administering matrilin-3-primed ASC spheroids with HA for patients with chronic discogenic LBP. Notably, six (75%) out of the eight patients showed significant improvements in both the VAS pain and ODI scores. T2-weighted MRI further revealed reduced HIZ or disc protrusion in half of these improved patients, with sustained pain relief over six months. These encouraging results advocate for progression to phase II randomized clinical trials.

## Figures and Tables

**Table 1:** Patient characteristics and characterization of cell products.

		Case Number							
		1	2	3	4	5	6	7	8
Sex (M/F)		M	M	M	M	M	F	M	M
Age (years)		54	46	32	64	38	52	51	50
BMI (kg/m^2^)		23.3	25.1	26.8	26.8	28.41	23.8	25.4	20.6
Hypertension (Y/N)		N	N	N	Y	N	N	N	N
Diabetes mellitus (Y/N)		N	N	N	N	N	N	N	N
Smoking history (Y/N)		N	N	Y	N	N	N	N	N
Duration of LBP (month)		120	36	37	11	60	12	49	48
Implanted disc level		L4/5	L4/5	L4/5	L4/5	L4/5	L4/5	L4/5	L4/5
Preoperative VAS		9	7	6	5	9	6	4	6
Preoperative ODI (%)		45	56	35	45	70	45	35	35
Preoperative modified Pfirrmann grade		Ⅴ	Ⅳ	Ⅵ	Ⅴ	Ⅳ	Ⅳ	Ⅲ	VII
Cell number in a vial (×10^7^)		1	1	1	1	1	1	1	1
Injected cell number with 400 μL of hyaluronic acid (×10^6^)		6.0	6.0	6.0	6.0	6.0	6.0	6.0	6.0
Cell viability (%)		91.5	95.86	96.55	95.8	96.55	92.76	96.46	95.9
Cell surface marker	CD44 (%)	87.2	100	100	99.9	99.7	100	99.6	98.4
	CD73 (%)	97.9	98.9	99	98.7	96.8	87.4	94.1	94.4
	CD45 (%)	1.6	1.4	0.8	1	0.6	0.5	0.8	0.5

M: male; F: female; BMI: body mass index; Y: yes; N: No; LBP: low back pain; VAS: visual analogue scale; ODI: Oswestry Disability Index.

**Table 2:** VAS and ODI outcomes according to time points.

Case No.	VAS (0–10 Point)					ODI (%)				
	Pre	1 W	1 M	3 M	6 M	Pre	1 W	1 M	3 M	6 M
1	9	6	8	6	7	45	45	48	45	45
2	7	4	3	3	3	56	45	40	34	25
3	6	5	4	4	3	35	30	25	15	15
4	5	1	1	0	0	45	35	10	0	0
5	9	6	5	5	5	70	55	45	40	35
6	6	7	5	4	3	45	30	30	35	33
7	4	1	1	2	1	35	20	20	30	20
8	6	5	6	2	2	35	30	35	25	25

VAS: visual analogue scale; ODI: Oswestry Disability Index; Pre: Pre-treatment; W: week; M: month.

**Table 3:** Percentage VAS pain score and ODI score reduction from baseline.

Case No.	Percentage VAS Score Reduction				Percentage ODI Score Reduction			
	1 W	1 M	3 M	6 M	1 W	1 M	3 M	6 M
1	33	11	33	22	0	−7	0	0
2	43	57	5	57	20	29	39	55
3	17	33	33	50	14	29	57	57
4	80	80	100	100	22	78	100	100
5	33	44	44	44	21	36	43	50
6	−17	17	33	50	33	33	22	27
7	75	75	50	75	43	43	14	43
8	17	0	67	67	14	0	29	29

VAS: visual analogue scale; ODI: Oswestry Disability Index; W: week; M: month; minus (−): Increase.

**Table 4:** Radiological outcomes according to time points.

Case No.	Modified Pfirrmann Grade		
	Baseline	1 M	6 M
1	V	V	V
2	IV	IV	IV
3	VI	VI	VI
4	V	V	V
5	IV	IV	IV
6	IV	IV	IV
7	III	III	III
8	VII	VI	VI

**Table 5:** Clinical trials using mesenchymal stem cells for chronic discogenic low back pain.

Year, Author	Stem Cells	Cell Number	Case No.	FU (Months)	Finding
2011 Orozco L et al. [35]	Autologous BM	23 ± 5 × 10^6^	10	12	VAS, ODI Improvement in VAS, ODI; improvement of water content on MRI
2013 Pang et al. [47]	Umbilical Cord Mesenchymal Stem Cells		2	24	The VAS and ODI scores decreased obviously during a 2-year follow-up period
2014 Pettine KA et al. [40]	Autologous BM concentrate	121 × 10^6^ total nucleated cell/ml	26	12	Improvement in VAS, ODI. Hgher concentration MSC injection patients versus lower concentration. Improvement of at least 1 grade in Modified Pfirrmann Score in 8 of 20 at 12 months
2015 Pettine KA et al. [44]	Autologous BM concentrate	121 × 10^6^ total nucleated cell/ml	Same cohort as above	24	Reduction in ODI and VAS scores endured at 24 months
2017 Pettine KA et al. [45]	Autologous BM concentrate	121 × 10^6^ total nucleated cell/ml	Same cohort as above	36	Reduction in ODI and VAS scores endured at 36 months
2015 Mochida J et al. [41]	Coculture of NP cells with autologous BM	Activated NP cells: 10^6^	9	36	JOA scoring system improvement. No injected disc showed worsening degeneration on MRI.
2016 Elabd et al. [42]	Autologous bone marrow-derived MSCs	15.8–37.3 × 10^6^ cells/ disc	5	48–72	No adverse events related to MSC Injection. Majority reported an overall improvement in QoL, strength, and mobility. Effect independent of cell dose
2017 Kumar et al. [16]	Autologous ASCs in HA carrier	2 × 10^7^ 4 × 10^7^	10	12	Improvement in VAS, ODI, SF-36 (n = 6); improvement of water content on diffusion MRI (n = 3)
2017 Noriega DC et al. [46]	Allogenic BM MSCs	25 × 10^6^	24 Cohort study with control group	12	Improvement in VAS and ODI. Improvement in Pfirrmann disc degeneration grade at 12 months
2017 Centeno et al. [43]	autologous bone marrow-derived MSCs	2.3 × 10^7^ cells/disc range 1.73–45 × 106	33	72	Improvement in NPS, FRI scores.
2021 Amirdelfan et al. [37]	Allogenic MPCs in HA carrier	6 × 10^6^ 1.8 × 10^7^	100	36	Improvement in VAS, ODI, SF-36, Work Productivity and Activity Index MRI
2022 Bates et al. [38]	Autologous ASCs	1 × 10^7^	9	12	Improvement in pain (78%); Increased work capacity (56%); Reduced analgesic medication (33%); Improvements in EQ-5D and ODI,
2022 Atluri et al. [39]	Autologous BM-MSCs	2.1 × 10^6^	80	12	Improvement in ODI, pain, and other parameters (EQ-5D-3L, GMH, and GPH).
Present study	Autologous ASCs spheroids with matrillin-3-priming	6 × 10^6^	8	6	Improvement in VAS and ODI Improvement in MRI: reduction of hyperintensity zone and central disc protrusion

FU: follow-up; M: months; No: number; ASC: adipose-derived mesenchymal stem cell; BM-MSC: bone marrow-derived mesenchymal stem cell; MPC: mesenchymal precursor cell; HA: hyaluronic acid; VAS: visual analogue scale; ODI: Oswestry Disability Index; SF: Short Form; MRI: magnetic resonance imaging; EQ-5D-3L: EuroQOL 5-Dimensional Questionnaire; QoL: quality of life; FRI:functional rating index; JOA: Japanese Orthopedic Association score; NPS:numeric pain score (0–10, 0 = no pain, 10 = worst possible pain); GMH: Global Mental Health; GPH: Global Physical Health.

**Table 6:** Assessment schedule.

	SV	V1	V2	V3	V4	V5	V6
Day	−42	−21	0	7	30	90	180
Time window (days)	NA	±3	±1	±1	±7	±7	±7
Informed consent	X						
Physical examination	X	X	X	X	X	X	X
Vital signs	X	X	X	X	X	X	X
Medical history	X						
Laboratory assessments	X		X	X	X	X	X
VAS	X	X	X	X	X	X	X
ODI	X	X	X	X	X	X	X
Lumbar spine X-ray	X				X		X
Lumbar spine MRI	X				X		X
Liposuction		X					
Stem cell transplantation			X				
AE assessment				X	X	X	X
SAE assessment				X	X	X	X

SV: screen visit; NA: not applicable; V: visit; VAS: visual analogue scale; ODI: Oswestry Disability Index; MRI: magnetic resonance imaging; AE: adverse event; SAE: severe adverse event.

**Table 7:** Inclusion and exclusion criteria.

Inclusion criteria
Age between 18 to 70 years
Chronic LBP that is unresponsive to at least 3 months of conservative treatment (i.e., medication, intensive physical therapy, and local anesthetic infiltration in facet joints or medial branches) Pretreatment (baseline) back pain intensity of 4/10 or higher on a visual analog scale (VAS) Pretreatment (Baseline) Oswestry Disability Index Questionnaire (ODI) score of 30 or higher
Diagnosis of chronic LBP based on clinical and MRI data (modified Pfirrmann grade III–VII at one or two levels based on T2-weighted MRI)
Able to comply with the protocol physically and mentally, able to adhere to the requirements of the protocol, and willing to voluntarily sign the informed consent form.
Exclusion criteria
Radiculopathy resulting from nerve compression at screening or baseline, and unilateral or bilateral leg pain with intensity greater than 50% of the intensity of the low back pain as measured on a VAS at screening or baseline. Prior lumbar spine surgery
Prior lumbar vertebral body fracture
Severely herniated disc or spinal stenosis requiring surgery
Degenerative changes in the lumbar as determined by Modic Changes Type 3
Evidence of dynamic instability on lumbar flexion extension, and Grade 2 or higher spondylolisthesis at the target disc at screening.
Significant underlying neurological condition
Pregnant, breastfeeding, or planning to become pregnant within 2 years
Evidence of a spinal infection on an MRI
Uncontrolled hypertension, uncontrolled diabetes, and other serious systemic diseases, such as cancer, an autoimmune disease, blood disease, and kidney disease

LBP: low back pain; IVD: intervertebral disc; MRI: magnetic resonance imaging.

## Data Availability

Data is contained within the article.
